# Epidemiology, temporal trends and co-infection of 13 respiratory pathogens in 32,125 children: a 3-year retrospective study

**DOI:** 10.3389/fped.2026.1884107

**Published:** 2026-08-14

**Authors:** Yu Zhang, Siyu Wang, Xiaoming Huang

**Affiliations:** 1 Department of General Pediatrics, West China Second University Hospital, Sichuan University, Chengdu, Sichuan, China; 2 Key Laboratory of Birth Defects and Related Diseases of Women and Children (Sichuan University), Ministry of Education, Chengdu, Sichuan, China

**Keywords:** acute respiratory tract infections (ARTIs), children, co-infection, epidemiological characteristics, multiplex PCR, respiratory pathogens

## Abstract

**Background:**

Pediatric acute respiratory tract infections bring heavy global disease burden. Clarifying post-pandemic epidemiological features guides clinical management and public health prevention.

**Methods:**

This retrospective study enrolled patients under 18 years receiving 13-pathogen multiplex PCR at West China Second University Hospital from 2023 to 2025. We extracted laboratory data and calculated pathogen positivity, single/co-infection proportions. Stratified analyses by year, season, age and sex and statistical collation of co-infection patterns were completed.

**Results:**

In total, 32,125 children were included. The total pathogen positive rate reached 63.4%, including 49.8% single infection and 13.6% co-infection dominated by dual infection. Human rhinovirus (22.9%) ranked first, followed by respiratory syncytial virus (15.2%). Pathogens varied obviously across seasons and ages: HRV surged in spring and autumn; parainfluenza, adenovirus and coronavirus prevailed in summer; syncytial virus and influenza peaked in winter. Respiratory syncytial virus mainly affected infants; rhinovirus and parainfluenza were prevalent in toddlers and preschoolers; Mycoplasma pneumoniae rose with age and peaked among school-age children. Dual infection occupied nearly all co-infections; the top non-influenza combinations were HRSV–HRV and HRV–HAdV. Infections with three or more pathogens were rare.

**Conclusions:**

HRV and HRSV dominated pediatric respiratory pathogens with obvious seasonal and age clustering. Most co-infections occurred in winter and young kids. HRV detection requires careful explanation for potential asymptomatic carriage. Local epidemiological characteristics should inform age-and seasonal-targeted diagnosis and targeted prevention including HRSV prevention.

## 1 Introduction

Acute respiratory tract infections (ARTIs) remain the leading cause of pediatric morbidity and mortality worldwide, accounting for a substantial proportion of hospital admissions, outpatient visits, and emergency department consultations in children under 18 years of age (1). The etiological spectrum of pediatric ARTIs is broad and complex, encompassing a wide range of viral and atypical bacterial pathogens, most notably *Human rhinovirus* (HRV), *respiratory syncytial virus* (HRSV), *parainfluenza virus* (HPIV), *adenovirus* (HADV), influenza viruses, *human metapneumovirus* (HMPV), *human coronaviruses* (HCoV), *human bocavirus* (Boca), *Mycoplasma pneumoniae* (*MP*), and *Chlamydia species* (*Ch*) (2, 3). With the widespread application of multiplex polymerase chain reaction (PCR) assays in clinical practice, the detection of multiple pathogens has become increasingly feasible, revealing not only single infections but also frequent co-infection that may complicate clinical presentation and management (4, 5).

The epidemiology of respiratory pathogens in children exhibits pronounced variations by season, age group, and geographical region, and has been significantly perturbed by the coronavirus disease 2019 (COVID-19) pandemic and the subsequent implementation and relaxation of non-pharmaceutical interventions (NPIs) (6–8). During the pandemic, the circulation of many common respiratory viruses was profoundly suppressed, only to resurge in an atypical manner once restrictions were lifted, leading to shifted seasonality, altered age distributions, and the reappearance of pathogens with unpredictable patterns (9–11). In the post-pandemic era from 2023 to 2025, continuous surveillance of the full spectrum of respiratory pathogens is therefore critical to understanding the evolving landscape of pediatric ARTIs, optimizing empirical treatment strategies, and guiding public health policy (3, 12).

Despite numerous studies on individual or limited groups of respiratory pathogens in children, comprehensive investigations that simultaneously examine a large panel of 13 respiratory pathogens—including seasonal coronaviruses, Boca, different influenza A subtypes, and atypical bacteria—across a large and age-stratified pediatric population remain scarce. Furthermore, detailed data on co-infection patterns, their population distribution, and temporal dynamics over multiple consecutive years are essential to fill the existing knowledge gaps. In this context, we conducted a large-scale, retrospective analysis of 32,125 pediatric patients who presented to our institution between 2023 and 2025, encompassing inpatients, outpatients, and emergency patients. The aim of this study was to comprehensively delineate the overall epidemic situation, pathogen spectrum composition, annual and seasonal temporal trends, sex and age-specific detection rates, and the detailed landscape of single and multiple co-infection for 13 respiratory pathogens. These findings are intended to provide a robust epidemiological foundation for informing prevention and control strategies for pediatric respiratory infections in the current post-pandemic period.

## 2 Materials and methods

### 2.1 Ethics statement

This retrospective study was conducted in accordance with the Declaration of Helsinki (1964) and its subsequent amendments, and the research protocol was approved by the Ethics Committee of West China Second University Hospital, Sichuan University (Approval No.: 151). The informed consent form was also approved by the hospital's ethics committee. The ethical review approval document is provided in Supplementary Figure S1.

### 2.2 Study participants

#### 2.2.1 The inclusion criteria were as follows

Children under 18 years old who underwent nucleic acid testing for 13 respiratory pathogens at West China Second University Hospital, Sichuan University between January 1, 2023 and December 31, 2025. Specimens were nasopharyngeal swabs or throat swabs, with complete clinical data.

#### 2.2.2 The exclusion criteria were as follows

(a) Children with incomplete basic information, including lack of one-time visit ID, or missing age or sex information. A “one-time visit ID” was defined as the unique system-assigned identifier corresponding to a single patient visit, within which all pathogen tests were ordered. This ID was used to define an independent testing event, calculate detection rates, and determine co-infection identified from the same visit.
(b) For patients with multiple test records under the same one-time visit ID, only the first test result was retained to ensure independent analysis.

#### 2.2.3 Basic information

One-time visit ID, Sex, age, visit type, test date and results of nucleic acid tests for 13 respiratory pathogens were clearly documented. Children were stratified into six age categories: newborns (0–28 days), infants (29 days to <1 year), toddlers (1 to <3 years), preschoolers (3 to <6 years), school-aged children (6 to <12 years), and adolescents (12–18 years). Seasons were demarcated according to the Northern Hemisphere meteorological calendar: spring (March–May), summer (June–August), autumn (September–November), and winter (December–February).

### 2.3 Detection methods and pathogen species

#### 2.3.1 Detection technique

The nucleic acids of specimens were extracted by the automated nucleic acid extraction system (Nucleic Acid Extraction or Purification Kit, HEALTH GENETECH, Ningbo, China). Testing for 13 respiratory pathogens was performed according to the manufacturer's instructions using a multiplex PCR-capillary electrophoresis panel (Multiplex detection of 13 respiratory tract pathogens with PCR capillary electrophoresis, HEALTH GENETECH, Ningbo, China). As specified in the manufacturer's kit manual, this multiplex detection assay only enables subtype identification of *influenza A virus* and lacks subtyping capacity for all other respiratory pathogens. Restricted by the detection methodology, we only documented the positive results of different influenza A subtypes in this study. For the rest pathogens, only overall positive rates were reported, and no detection data of respective subtypes were collected.

#### 2.3.2 Respiratory pathogens

*Human rhinovirus* (HRV), *human bocavirus* (Boca), *Mycoplasma pneumoniae* (*MP*), *human parainfluenza virus* (HPIV), *human coronavirus* (HCoV), *human respiratory syncytial virus* (HRSV), *influenza A virus* subtype H3N2 (H3N2), *influenza A virus* (InfA), *2009* pandemic *influenza A* (H1N1*) virus* (InfA.H1N1.2009), *human metapneumovirus* (HMPV), *human adenovirus* (HADV), *Chlamydia species* (*Ch*), and *influenza B virus* (InfB).

#### 2.3.3 Criteria for positivity

A positive result was determined according to the kit instructions.

Co-infection was defined as the simultaneous detection of two or more pathogens.

#### 2.3.4 Quality control and longitudinal consistency

Batch validity: Must meet kit QC criteria; otherwise invalid and retest.

Positive control: 16 peaks (13 pathogen + 3 IC) all detected, heights > PosS, absolute fragment length error < 1.5 nt.

Negative control: huDNA and IC peaks detected, heights > PosS; no pathogen peaks; length error < 1.5 nt.

Standards: Store protected from light, avoid repeated freeze-thaw. Re-run standards after instrument relocation, capillary replacement, or optical maintenance to set a new baseline.

Sample preparation: Follow SOP strictly. Vortex throat swabs 10 s; use the same model of extraction kit and automated instrument throughout to minimize operational variability.

### 2.4 Specimen collection

Trained clinicians and nurses collected pharyngeal swab samples from children following standard operating procedures. Preferentially-selected flocked swabs were used, and the oral cavity was cleaned with sterile saline if required. Each child tilted their head back and opened the mouth to fully expose pharyngeal tissues; caregivers restrained younger children to ensure sampling safety. The swab was rubbed against bilateral tonsils (2–3 times per side) and posterior pharyngeal wall (2–3 times) with moderate force while avoiding the tongue and oral mucosa. Afterwards, swab tips were promptly placed into cell-preservation liquid. All specimens underwent pathogen testing within 24-hours post-collection.

### 2.5 Data collection and organization

#### 2.5.1 Data source

Data were obtained from the hospital electronic medical record database. Variables collected included one-time visit ID, sex, age, visit type, test date, and results of the 13 respiratory pathogens.

#### 2.5.2 Data processing

Data were entered and organized using Microsoft Excel and double-checked by two independent researchers. Invalid data were excluded.

### 2.6 Statistical analysis

#### 2.6.1 Descriptive statistics

Count data were presented as numbers (*n*) and percentages (%), while measurement data (e.g., age) were expressed as mean and standard deviation.

#### 2.6.2 Between-group comparisons

The chi-square test was used to compare pathogen positive rates among sex, age, season, and year subgroups. For comparisons that did not meet the chi-square test criteria, Fisher's exact test was employed.

#### 2.6.3 Temporal trend analysis

The Cochran-Armitage trend chi-square test was applied to analyze the changing trends of each pathogen's positive rate over the 3 years.

#### 2.6.4 Co-infection analysis

The incidence of co-infection was analyzed.

Statistical analyses were performed using R4.2.2 (http://www.R-project.org, The R Foundation) and Free Statistics software version 2.4. A *P* value <0.05 was defined as statistically significant.

## 3 Results

### 3.1 Basic characteristics of 32,125 children

**Figure 1:**
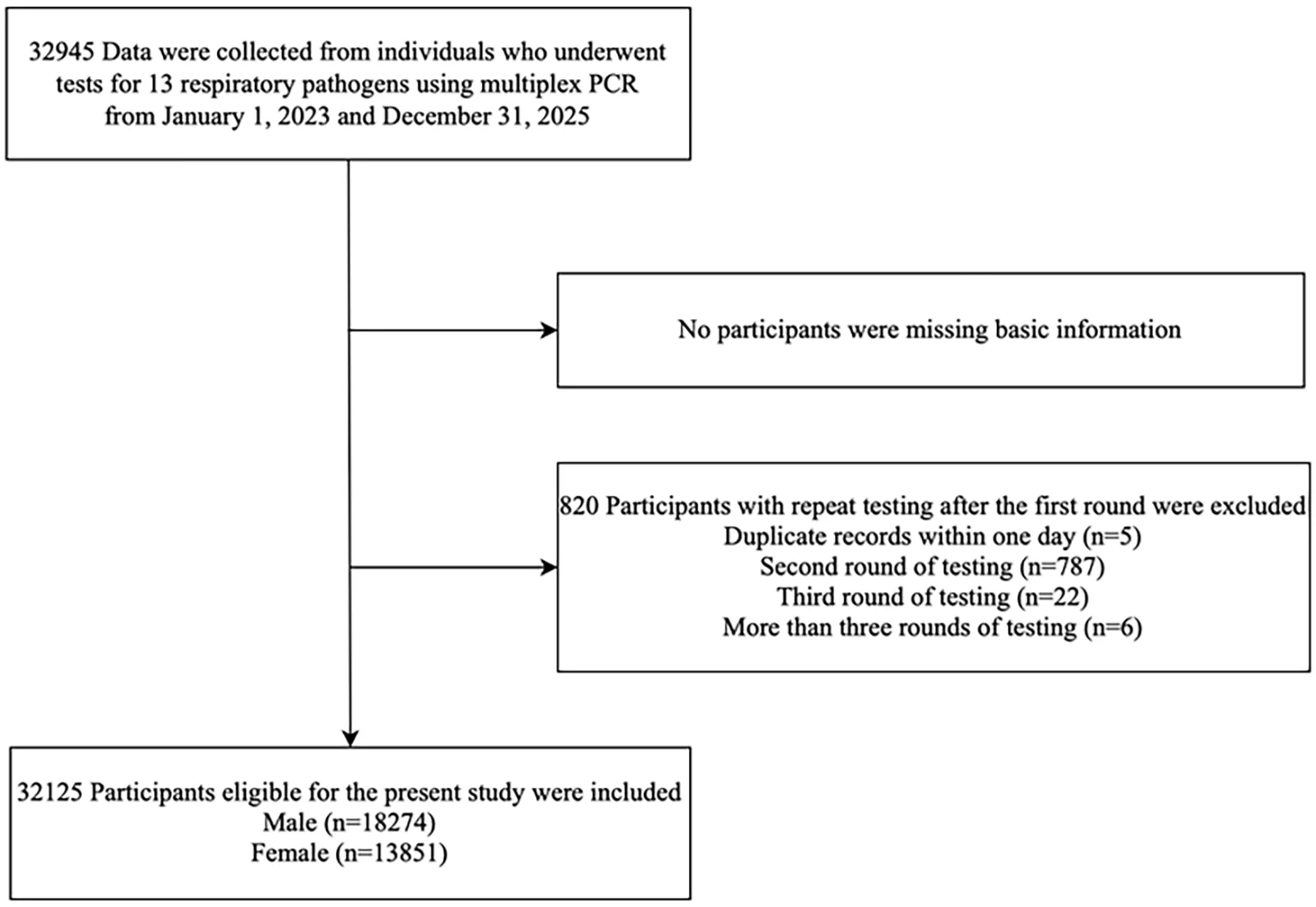
Flowchart of participant enrollment, exclusion criteria, and final analytical cohort (*n* = 32,125).

General characteristics: A total of 32,125 children were enrolled. The child participant selection process is described in detail in Figure 1. We summarized the counts and corresponding percentages stratified by sex (male/female), age groups (newborns/infants/toddlers/preschoolers/school-aged children/adolescents), visit type (IP/OP/EP), year (2023/2024/2025), and season (spring/summer/fall/winter). Detailed results are presented in Table 1.

**Table 1:** Demographic and clinical characteristics of 32,125 children stratified by sex, age, year, season, and visit type.

Characteristics	*n*	%
Total	32,125	100
Sex		
Male	18,274	56.9
Female	13,851	43.1
Year		
2023	8,073	25.1
2024	13,701	42.6
2025	10,351	32.2
Season		
Spring	7,713	24.0
Summer	7,559	23.5
Fall	8,204	25.5
Winter	8,649	26.9
Age groups		
Newborns (0–28 days)	1,520	4.7
Infants (29 days–<1 year)	7,505	23.4
Toddlers (1–<3 years)	7,781	24.2
Preschoolers (3–<6 years)	7,424	23.1
School-aged children (6–<12 years)	6,360	19.8
Adolescents (12–18 years)	1,535	4.8
Visit type		
IP (inpatient)	19,984	62.2
OP (outpatient)	2,550	7.9
EP (emergency patient)	9,591	29.9

Percentages were rounded to one decimal place; column sums may not equal 100% due to rounding.

### 3.2 Overall epidemic situation of 13 respiratory pathogens

#### 3.2.1 Overall positive rate

A total of 20,381 positive children were identified among 32,125 children, corresponding to a positive rate of 63.4%.

#### 3.2.2 Pathogen spectrum composition

**Figure 2:**
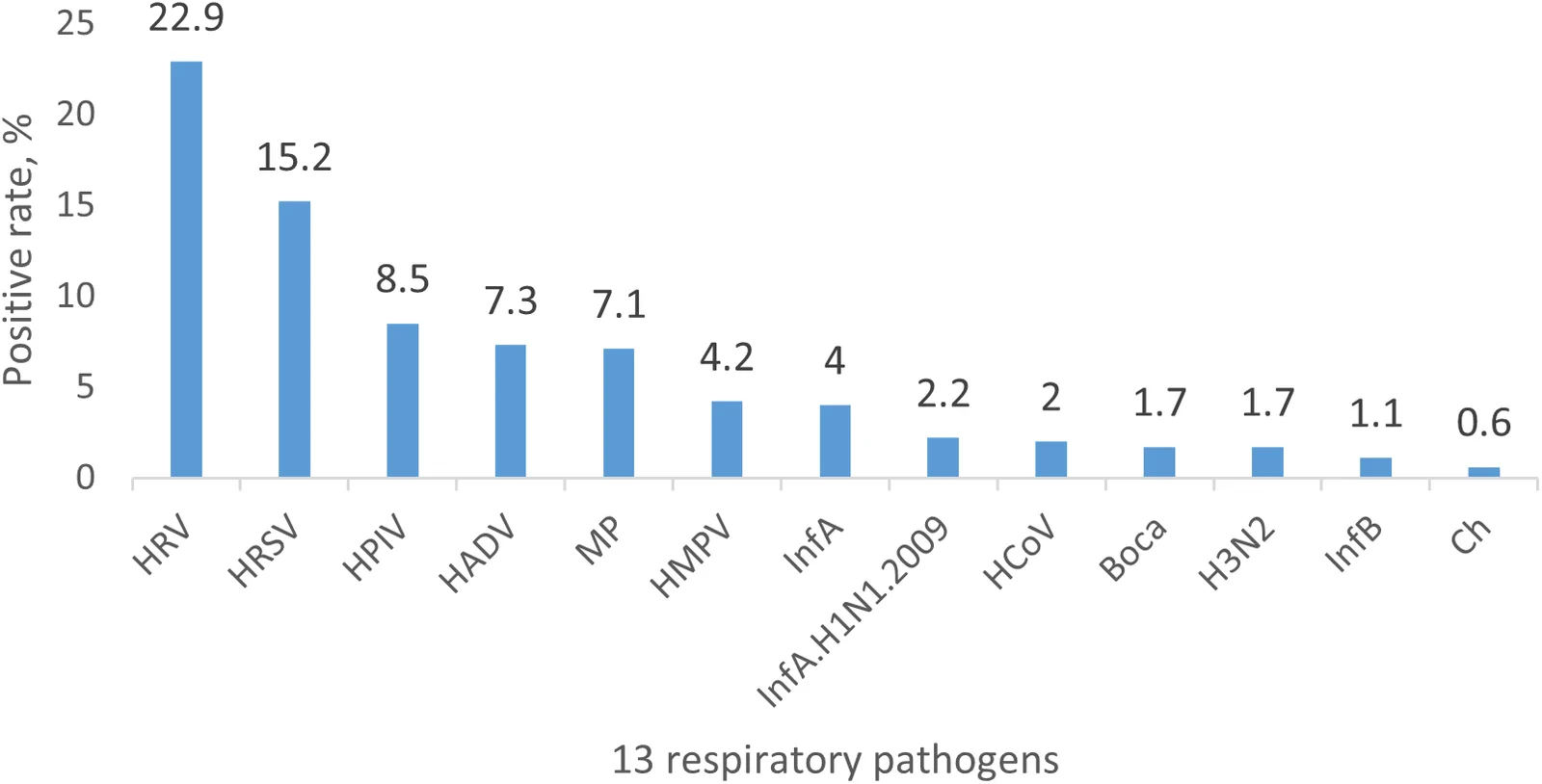
Overall positivity rates of 13 respiratory pathogens among 32,125 pediatric patients, 2023–2025 (%). HRV, *Human rhinovirus*; Boca, *human bocavirus*; *MP*, *Mycoplasma pneumoniae*; HPIV, *human parainfluenza virus*; HCoV, *human coronavirus*; HRSV, *human respiratory syncytial virus*; H3N2, *influenza A virus* subtype H3N2; InfA, *influenza A virus*; InfA.H1N1.2009, *2009* pandemic *influenza A* (H1N1) virus; HMPV, *human metapneumovirus*; HADV, *human adenovirus*; *Ch*, *Chlamydia species*; and InfB, *influenza B virus*.

Among the 32,125 children, HRV and HRSV were the predominant pathogens, with infection rates of 22.9% and 15.2%, respectively, followed by HPIV (8.5%), HADV (7.3%), *MP* (7.1%), HMPV (4.2%), and InfA (4.0%). The remaining pathogens—including HCoV, H3N2, Boca, InfB, InfA.H1N1.2009, and *Ch*—each accounted for ≤2.2% of infections (Figure 2).

#### 3.2.3 Distribution of single infections and co-infection

Of the 32,125 specimens analyzed, 36.6% were pathogen-negative, 49.8% harbored a single pathogen, and 13.6% showed co-infections, among which double, triple, quadruple, and quintuple infections accounted for 12.2%, 1.3%, 0.1%, and <0.1%, respectively (Table 2).

**Table 2:** Overall distribution of pathogen-negative, single-infection, and co-infection cases (dual to quintuple).

Detection of pathogens	*n*	%
No pathogen infection	11,744	36.6
Single pathogen infection	16,003	49.8
Double pathogen infection	3,919	12.2
Triple pathogen infection	426	1.3
Quadruple pathogen infection	31	0.1
Quintuple pathogen infection	2	<0.1%

### 3.3 Temporal trend analysis of respiratory pathogens

#### 3.3.1 Annual change trend

**Figure 3:**
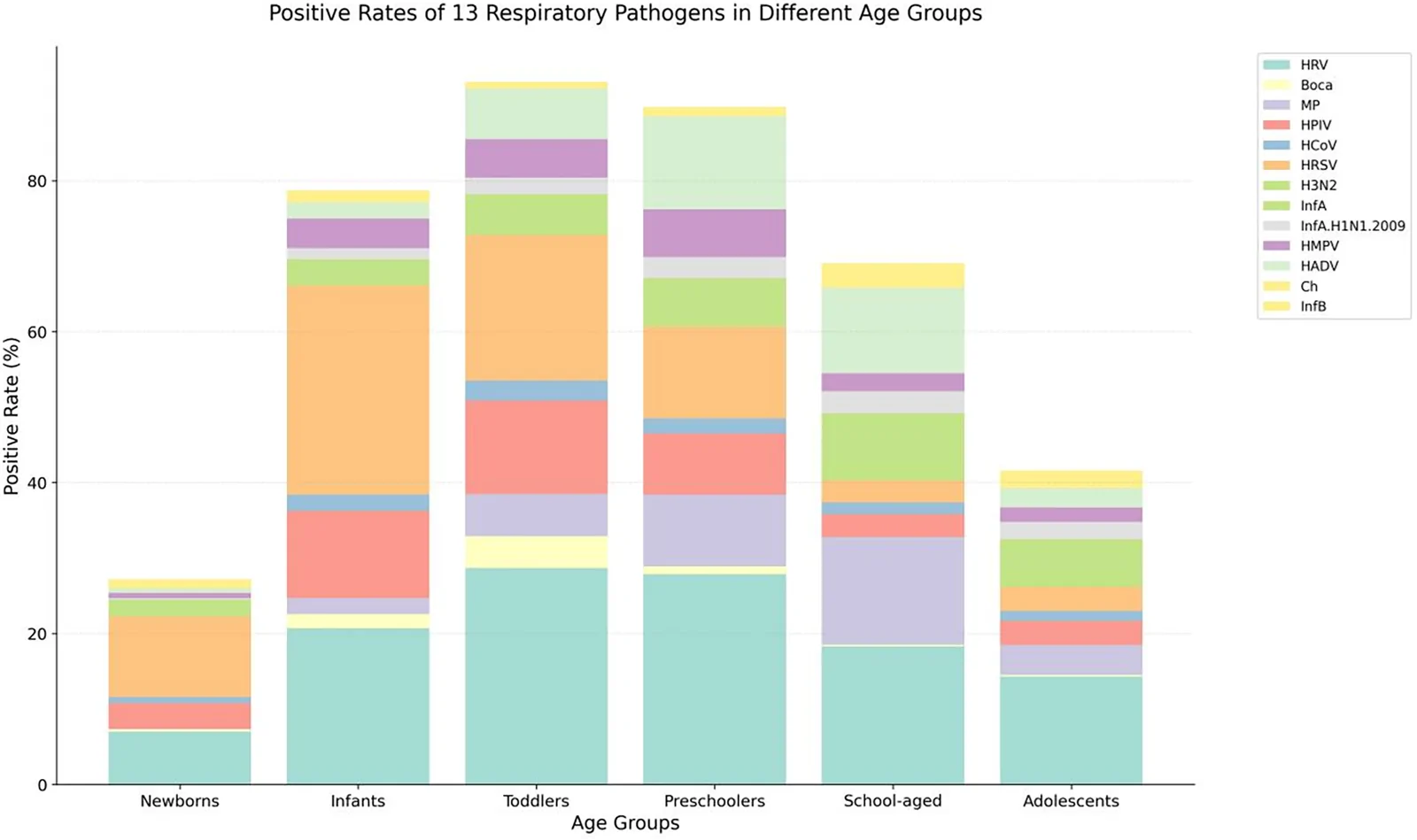
Annual trends in positivity rates of 13 respiratory pathogens from 2023 to 2025. HRV, *Human rhinovirus*; Boca, *human bocavirus*; *MP*, *Mycoplasma pneumoniae*; HPIV, *human parainfluenza virus*; HCoV, *human coronavirus*; HRSV, *human respiratory syncytial virus*; H3N2, *influenza A virus* subtype H3N2; InfA, *influenza A virus*; InfA.H1N1.2009, *2009* pandemic *influenza A* (H1N1) virus; HMPV, *human metapneumovirus*; HADV, *human adenovirus*; *Ch*, *Chlamydia species*; and InfB, *influenza B virus*.

From 2023 to 2025, HRV consistently dominated, peaking at 25.3% (2024) and leveling at 24.6% (2025). HRSV followed a U-shaped pattern (15.1% → 11.2% → 20.6%), whereas *MP* declined steadily from 12.0% to 1.3%. HPIV and HADV showed moderate fluctuations, with HADV peaking at 12.9% in 2024. All other pathogens remained below 5% positivity with minimal year-to-year change (Figure 3, Supplementary Table S1).

#### 3.3.2 Seasonal distribution characteristics

Among the 32,125 children enrolled, the four seasonal cohorts included spring (*n* = 7,713), summer (*n* = 7,559), fall (*n* = 8,204), and winter (*n* = 8,649). All 13 pathogens exhibited significant seasonal variation (*P* < 0.05), with distinct predilection patterns: HRV predominated in spring and fall (27.4% and 27.9%); Boca, HPIV, HCoV, and HADV peaked in summer—notably HPIV at 17.3%; HRSV, along with influenza-related pathogens (H3N2, InfA, InfA.H1N1.2009, HMPV, InfB), peaked in winter, with HRSV reaching 21.9%. *MP* and *Ch* showed relatively stable distributions across seasons (*MP*: 7.1% overall, 8.3% in summer; *Ch*: 0.4%–0.7%). HPIV and HRSV exhibited the most pronounced seasonal variation (*χ*^2^ = 1,121.71 and 1,122.46, respectively), serving as the primary etiological agents of summer and winter respiratory infections, respectively (Table 3).

**Table 3:** Seasonal positivity rates of 13 respiratory pathogens, with *χ*^2^ test results for inter-seasonal comparisons.

Variables	Total (*n* = 32,125)	Spring (*n* = 7,713)	Summer (*n* = 7,559)	Fall (*n* = 8,204)	Winter (*n* = 8,649)	*P*	Statistic
HRV, *n* (%)	7,341 (22.9)	2,115 (27.4)	1,548 (20.5)	2,288 (27.9)	1,390 (16.1)	<0.001	459.12
Boca, *n* (%)	562 (1.7)	45 (0.6)	343 (4.5)	121 (1.5)	53 (0.6)	<0.001	471.51
*MP*, *n* (%)	2,267 (7.1)	398 (5.2)	626 (8.3)	591 (7.2)	652 (7.5)	<0.001	62.92
HPIV, *n* (%)	2,725 (8.5)	619 (8)	1,308 (17.3)	525 (6.4)	273 (3.2)	<0.001	1,121.71
HCoV, *n* (%)	647 (2.0)	95 (1.2)	243 (3.2)	177 (2.2)	132 (1.5)	<0.001	90.43
HRSV, *n* (%)	4,886 (15.2)	1,292 (16.8)	277 (3.7)	1,426 (17.4)	1,891 (21.9)	<0.001	1,122.46
H3N2, *n* (%)	559 (1.7)	88 (1.1)	3 (0)	194 (2.4)	274 (3.2)	<0.001	265.88
InfA, *n* (%)	1,295 (4.0)	279 (3.6)	77 (1)	256 (3.1)	683 (7.9)	<0.001	532.42
InfA.H1N1.2009, *n* (%)	718 (2.2)	186 (2.4)	73 (1)	56 (0.7)	403 (4.7)	<0.001	379.99
HMPV, *n* (%)	1,349 (4.2)	264 (3.4)	116 (1.5)	221 (2.7)	748 (8.6)	<0.001	616.77
HADV, *n* (%)	2,355 (7.3)	426 (5.5)	783 (10.4)	593 (7.2)	553 (6.4)	<0.001	150.41
*Ch*, *n* (%)	203 (0.6)	55 (0.7)	31 (0.4)	55 (0.7)	62 (0.7)	0.048	7.92
InfB, *n* (%)	346 (1.1)	51 (0.7)	2 (0)	40 (0.5)	253 (2.9)	<0.001	394.86

HRV, *Human rhinovirus*; Boca, *human bocavirus*; *MP*, *Mycoplasma pneumoniae*; HPIV, *human parainfluenza virus*; HCoV, *human coronavirus*; HRSV, *human respiratory syncytial virus*; H3N2, *influenza A virus* subtype H3N2; InfA, *influenza A virus*; InfA.H1N1.2009, *2009* pandemic *influenza A* (H1N1) virus; HMPV, *human metapneumovirus*; HADV, *human adenovirus*; *Ch*, *Chlamydia species*; and InfB, *influenza B virus*.

### 3.4 Population distribution of respiratory pathogens

HRV was more frequently detected in males than females (24.3% vs. 21.0%, *χ*^2^ = 48.72, *P* < 0.001), as was Boca (2.0% vs. 1.5%, *χ*^2^ = 11.99, *P* < 0.001) and HRSV (15.6% vs. 14.7%, *χ*^2^ = 3.99, *P* = 0.046); *MP* was the only pathogen significantly higher in females (7.4% vs. 6.8%, *χ*^2^ = 4.56, *P* = 0.033). No significant sex differences were observed for the remaining nine pathogens (all *P* > 0.05).

**Figure 4:**
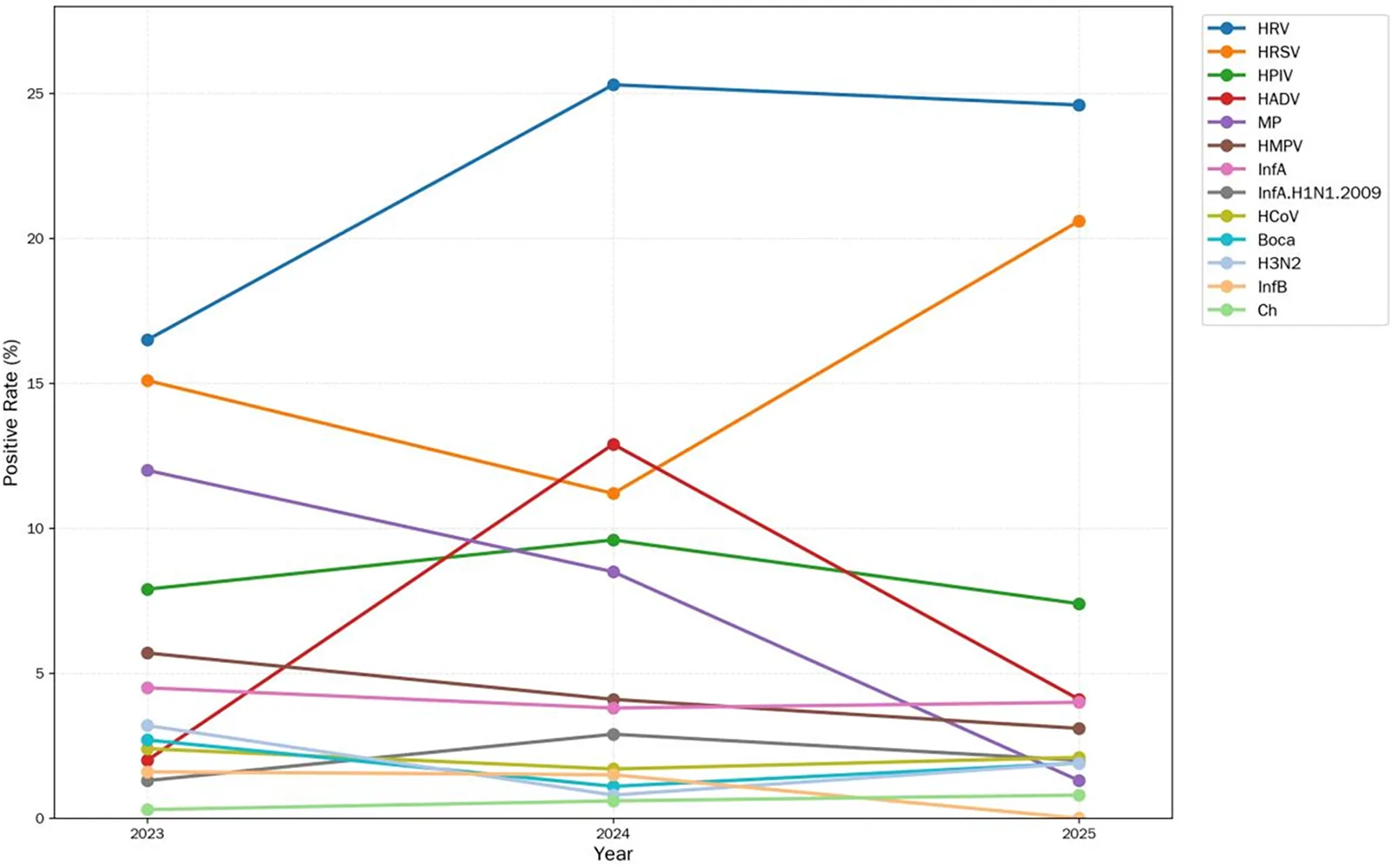
Positive rates of 13 respiratory pathogens across different pediatric age groups. newborns (0–28 days), infants (29 days to <1 year), toddlers (1 to <3 years), preschoolers (3 to <6 years), school-aged children (6 to <12 years), and adolescents (12–18 years). HRV, *Human rhinovirus*; Boca, *human bocavirus*; *MP*, *Mycoplasma pneumoniae*; HPIV, *human parainfluenza virus*; HCoV, *human coronavirus*; HRSV, *human respiratory syncytial virus*; H3N2, *influenza A virus* subtype H3N2; InfA, *influenza A virus*; InfA.H1N1.2009, *2009* pandemic *influenza A* (H1N1) virus; HMPV, *human metapneumovirus*; HADV, *human adenovirus*; *Ch*, *Chlamydia species*; and InfB, *influenza B virus*.

All 13 pathogens exhibited significant age-group differences (all *P* < 0.001, Table 4, Figure 4). HRV peaked in toddlers (28.7%) and preschoolers (27.9%); *MP* increased with age, peaking in school-aged children (14.3%); HRSV predominated in infants (27.7%) and declined progressively with age; HPIV, Boca, and HADV showed peaks in toddlers, infants, and preschoolers, respectively. HCoV, H3N2, InfA, InfA.H1N1.2009, HMPV, *Ch*, and InfB were detected predominantly in young and school-aged children but remained low in newborns and adolescents.

**Table 4:** Comparison of pathogen positive rates among different sex and age groups.

Respiratory pathogen	Total (*n* = 32,125)	Female (*n* = 13,851)	Male (*n* = 18,274)	*P*	Statistic	Newborns (*n* = 1,520	Infants (*n* = 7,505	Toddlers (*n* = 7,781	Preschoolers (*n* = 7,424	School-aged children (*n* = 6,360	Adolescents (*n* = 1,535	*P*	Statistic
HRV, *n* (%)	7,341 (22.9)	2,905 (21)	4,436 (24.3)	<0.001	48.72	107 (7.0)	1,550 (20.7)	2,230 (28.7)	2,072 (27.9)	1,162 (18.3)	220 (14.3)	<0.001	631.67
Boca, *n* (%)	562 (1.7)	202 (1.5)	360 (2)	<0.001	11.99	5 (0.3)	144 (1.9)	325 (4.2)	72 (1)	13 (0.2)	3 (0.2)	<0.001	421.99
*MP*, *n* (%)	2,267 (7.1)	1,026 (7.4)	1,241 (6.8)	0.033	4.56	2 (0.1)	159 (2.1)	436 (5.6)	702 (9.5)	907 (14.3)	61 (4)	<0.001	1,005.90
HPIV, *n* (%)	2,725 (8.5)	1,161 (8.4)	1,564 (8.6)	0.574	0.32	51 (3.4)	870 (11.6)	964 (12.4)	602 (8.1)	189 (3)	49 (3.2)	<0.001	603.42
HCoV, *n* (%)	647 (2.0)	281 (2)	366 (2)	0.870	0.03	12 (0.8)	161 (2.1)	206 (2.6)	147 (2)	101 (1.6)	20 (1.3)	<0.001	37.85
HRSV, *n* (%)	4,886 (15.2)	2,043 (14.7)	2,843 (15.6)	0.046	3.99	162 (10.7)	2,076 (27.7)	1,505 (19.3)	908 (12.2)	186 (2.9)	49 (3.2)	<0.001	1,997.09
H3N2, *n* (%)	559 (1.7)	245 (1.8)	314 (1.7)	0.732	0.12	15 (1)	78 (1)	118 (1.5)	133 (1.8)	186 (2.9)	29 (1.9)	<0.001	81.37
InfA, *n* (%)	1,295 (4.0)	552 (4)	743 (4.1)	0.716	0.13	18 (1.2)	190 (2.5)	300 (3.9)	341 (4.6)	379 (6)	67 (4.4)	<0.001	143.70
InfA.H1N1.2009, *n* (%)	718 (2.2)	304 (2.2)	414 (2.3)	0.671	0.18	3 (0.2)	112 (1.5)	174 (2.2)	206 (2.8)	187 (2.9)	36 (2.3)	<0.001	72.29
HMPV, *n* (%)	1,349 (4.2)	566 (4.1)	783 (4.3)	0.380	0.77	10 (0.7)	289 (3.9)	399 (5.1)	467 (6.3)	155 (2.4)	29 (1.9)	<0.001	216.48
HADV, *n* (%)	2,355 (7.3)	995 (7.2)	1,360 (7.4)	0.378	0.78	9 (0.6)	156 (2.1)	523 (6.7)	910 (12.3)	717 (11.3)	40 (2.6)	<0.001	871.86
*Ch*, *n* (%)	203 (0.6)	80 (0.6)	123 (0.7)	0.285	1.15	10 (0.7)	58 (0.8)	7 (0.1)	28 (0.4)	83 (1.3)	17 (1.1)	<0.001	97.88
InfB, *n* (%)	346 (1.1)	157 (1.1)	189 (1)	0.393	0.73	7 (0.5)	60 (0.8)	63 (0.8)	67 (0.9)	130 (2)	19 (1.2)	<0.001	74.38

HRV, *Human rhinovirus*; Boca, *human bocavirus*; *MP*, *Mycoplasma pneumoniae*; HPIV, *human parainfluenza virus*; HCoV, *human coronavirus*; HRSV, *human respiratory syncytial virus*; H3N2, *influenza A virus* subtype H3N2; InfA, *influenza A virus*; InfA.H1N1.2009, *2009* pandemic *influenza A* (H1N1) virus; HMPV, *human metapneumovirus*; HADV, *human adenovirus*; *Ch*, *Chlamydia species*; and InfB, *influenza B virus*.

### 3.5 Analysis of co-infection characteristics

#### 3.5.1 Overall characteristics of pathogen co-infection

A total of 4,378 co-infection cases were included for analysis. Stratified analyses demonstrated that the distribution of co-infections differed significantly by year, season, visit type and age group (all *P* < 0.001), whereas no statistically significant difference was found between sexes (*P* = 0.214).

In terms of annual distribution, co-infections were most prevalent in 2024 (47.5%), with comparable proportions in 2025 (26.2%) and 2023 (26.2%). Seasonally, the proportion peaked in winter (33.1%), followed by autumn (25.3%), spring (21.7%) and summer (20.0%). Classified by visit type, inpatients accounted for the largest share (48.1%), followed by emergency patients (40.6%), and outpatients had the lowest proportion (11.3%). Males made up 59.0% of co-infected children and females accounted for 41.0%, yet the difference lacked statistical significance. Across age groups, toddlers occupied the highest percentage (29.5%), followed by preschoolers (27.7%) and infants (21.5%); newborns had the minimum proportion (0.6%).

Stratified data of single infection and co-infection are detailed in Table 5. Information on specific pathogen co-infection combinations is summarized in Supplementary Table S2.

**Table 5:** Characteristics of children stratified by single infection vs. co-infection status, with comparisons across sex, year, season, age group, and visit type.

Variables	Total (*n* = 20,381)	Single infection (*n* = 16,003)	Co-infection (*n* = 4,378)	*P*	Statistic
Sex, *n* (%)				0.214	1.55
Female	8,533 (41.9)	6,736 (42.1)	1,797 (41.0)		
Male	11,848 (58.1)	9,267 (57.9)	2,581 (59.0)		
Year, *n* (%)				<0.001	64.17
2023	4,817 (23.6)	3,672 (22.9)	1,145 (26.2)		
2024	9,194 (45.1)	7,116 (44.5)	2,078 (47.5)		
2025	6,370 (31.3)	5,215 (32.6)	1,155 (26.4)		
Season, *n* (%)				<0.001	77.85
Spring	4,896 (24.0)	3,948 (24.7)	948 (21.7)		
Summer	4,479 (22.0)	3,603 (22.5)	876 (20.0)		
Fall	5,324 (26.1)	4,217 (26.4)	1,107 (25.3)		
Winter	5,682 (27.9)	4,235 (26.5)	1,447 (33.1)		
Age groups, *n* (%)				<0.001	75.17
Newborns	386 (1.9)	361 (2.3)	25 (0.6)		
Infants	4,857 (23.8)	3,916 (24.5)	941 (21.5)		
Toddlers	5,801 (28.5)	4,510 (28.2)	1,291 (29.5)		
Preschoolers	5,307 (26.0)	4,093 (25.6)	1,214 (27.7)		
School-aged children	3,526 (17.3)	2,736 (17.1)	790 (18.0)		
Adolescents	504 (2.5)	387 (2.4)	117 (2.7)		
Visit type, *n* (%)				<0.001	86.85
IP (inpatient)	11,024 (54.1)	8,919 (55.7)	2,105 (48.1)		
OP (outpatient)	1,876 (9.2)	1,382 (8.6)	494 (11.3)		
EP (emergency patient)	7,481 (36.7)	5,702 (35.6)	1,779 (40.6)		

#### 3.5.2 Co-infection combination analysis

Dual infection was the predominant form of co-infection. The top five co-infection combinations were InfA & InfA.H1N1.2009 (*n* = 599), HRSV & HRV (*n* = 496), InfA & H3N2 (*n* = 446), HRV & HAdV (*n* = 423), HRV & HPIV (*n* = 382). All other dual pathogen pairs exhibited relatively low detection frequencies.

**Figure 5:**
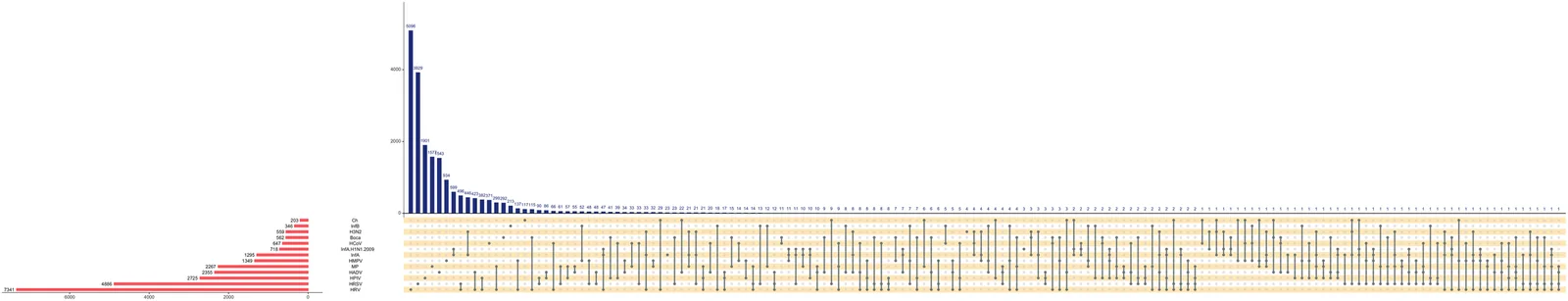
Distribution of dual and multiple co-infection patterns, with the top five dual pathogen combinations highlighted. A, double co-infection, b, triple co-infection, c, quadruples + quintuples co-infection. HRV, *Human rhinovirus*; Boca, *human bocavirus*; *MP*, *Mycoplasma pneumoniae*; HPIV, *human parainfluenza virus*; HCoV, *human coronavirus*; HRSV, *human respiratory syncytial virus*; H3N2, *influenza A virus* subtype H3N2; InfA, *influenza A virus*; InfA.H1N1.2009, *2009* pandemic *influenza A* (H1N1) virus; HMPV, *human metapneumovirus*; HADV, *human adenovirus*; *Ch*, *Chlamydia species*; and InfB, *influenza B virus*.

Infections with three or more pathogens showed low overall prevalence in this study population, and no dominant multi-pathogen combination was observed. Rare multi-pathogen cases were scattered among various triple, quadruple and quintuple pathogen combinations, suggesting concurrent infection with three or more pathogens is uncommon among pediatric patients in this cohort. Detailed distributions of mixed pathogens are presented in Figure 5.

## 4 Discussion

This large-scale pediatric surveillance spanning 2023–2025 (32,125 children) delineates a contemporary landscape of 13 respiratory pathogens, revealing an overall positivity of 63.4% and thereby confirming a substantial and sustained burden of respiratory infections in children. *Human rhinovirus* (HRV) and *human respiratory syncytial virus* (HRSV) were the dominant pathogens (22.9% and 15.2%, respectively), while single infection comprised 49.8% of cases and co-infection 13.6% (predominantly dual, 12.2%). These core figures underscore the necessity of prioritizing HRV and HRSV in routine diagnostic and preventive strategies.

Caution is required when interpreting the high detection rate of *Human rhinovirus* (HRV) observed in this study. Previous studies have demonstrated that HRV is frequently detected as an asymptomatic colonization in the upper respiratory tract of healthy children, with carriage rates ranging from 14% to 22% (13). Therefore, a proportion of the HRV-positive cases in our cohort may represent incidental detection rather than genuine symptomatic infection. This inherent limitation could lead to an overestimation of the clinical disease burden specifically attributable to HRV.

The lower positivity rates of *Mycoplasma pneumoniae* and influenza virus in this study, compared with many prior reports, may be explained by several factors. Methodologically, our multiplex nucleic acid panel detects only active pathogen shedding, whereas some earlier studies with higher rates used serological testing, which captures a broader diagnostic window (14). The sample source is another key determinant of the observed differences. Our specimens were nasopharyngeal swabs or throat swabs, whereas several studies with higher positivity rates utilized bronchoalveolar lavage fluid or sputum, which are known to have higher pathogen loads, especially for *Mycoplasma pneumoniae* (15, 16). In the 2023–2025 period, the widespread adoption of a “stepwise screening” strategy in routine clinical practice likely introduced a significant selection bias. In many settings, children with respiratory symptoms are initially tested using single-target nucleic acid tests for the most common pathogens, such as *Mycoplasma pneumoniae* and *influenza virus* (17). Only those who test negative or present with complex conditions subsequently undergo multiplex syndromic panel testing. Since our study exclusively enrolled patients who received multiplex testing, our cohort was effectively enriched for individuals who had already screened negative for these single pathogens. Consequently, the positivity rates of *M. pneumoniae* and *influenza virus* captured by our multiplex assay are lower than those reported in studies where single-target tests were universally applied as the primary diagnostic tool. This “clinical triaging” effect, rather than a true decline in circulation, may be a major driver of the discrepancy, especially for influenza virus during the post-pandemic resurgence period. This bias is particularly relevant for influenza virus. During the 2023–2025 period, influenza activity rebounded globally after the relaxation of COVID-19 restrictions, and single-target influenza rapid or PCR tests were heavily utilized. Many influenza-positive cases were diagnosed and managed without ever undergoing multiplex testing. The markedly reduced influenza positivity in our multiplex-tested population is therefore a predictable consequence of this diagnostic workflow, and does not necessarily contradict reports of high influenza prevalence from comprehensive, unbiased surveillance. Population differences also contribute: Our cohort comprised all children who underwent PCR testing, while many comparator studies focused on hospitalized or more severe cases (18).

### 4.1 Temporal trends

The 3-year trajectory of HRSV was marked by a pronounced rebound—from 15.1% in 2023, dipping to 11.2% in 2024, then rising sharply to 20.6% in 2025—particularly among infants and young children. Although the time since the lifting of COVID-19 non-pharmaceutical interventions cautions against an overly simplistic application of the “immune debt” concept, the observed pattern is fully consistent with it: prolonged reductions in viral exposure expanded the susceptible pediatric population, fueling a delayed, amplified resurgence once social contacts normalized (19). The transient dip observed in 2024 can be further understood by considering the interplay between post-pandemic dynamics and the natural biennial periodicity of HRSV. Following a large-scale outbreak in 2023—likely representing the first post-lockdown surge—a substantial proportion of previously unexposed infants and toddlers may have been infected, temporarily reducing the pool of susceptible hosts in 2024. This “depletion effect,” common in HRSV biennial cycles, typically results in a milder season, followed by a resurgence as new birth cohorts accumulate susceptibility (20). Additionally, local factors such as residual behavioral precautions, meteorological variations, or shifts in healthcare-seeking behavior during that specific year could have contributed to the reduced circulation, though these remain speculative without dedicated environmental or mobility data (21). This shift decouples current HRSV seasonality from historical fixed schedules for prophylaxis, arguing for flexible, surveillance-driven timing of monoclonal antibody administration to align with earlier and more intense winter peaks (22, 23).

In stark contrast, *Mycoplasma pneumoniae* (*MP*) prevalence fell progressively from 12.0% to 1.3%. Plausible contributors include post-epidemic depletion of susceptible hosts, altered healthcare-seeking behavior, natural cyclicity of *MP* epidemics and due to the widespread use of antibiotics post-pandemic, some patients had already received treatment prior to testing, resulting in the pathogen load falling below the detection limit (24–27). Given *MP*'s known cyclicity and its predilection for school-aged children (14.3% in this group), sustained laboratory-based surveillance remains essential to detect a future resurgence early (28). In parallel, antimicrobial stewardship must be refined: despite the declining prevalence, the reality of macrolide resistance cannot be ignored. Empiric macrolides should be reserved for children with high clinical probability—gradual-onset dry cough, school age, autumn–winter presentation (29, 30). Testing for resistance markers or the use of alternative agents should be considered in settings where resistance is prevalent (31). HRV maintained the highest and most stable positivity across all three years, reinforcing its role as a perennial driver of pediatric illness that co-circulates with seasonal peaks of other viruses and should be a constant consideration in diagnostic panels and co-infection assessments (32).

### 4.2 Seasonal characteristics

Clear and statistically robust seasonal patterns emerged for most pathogens (large effect sizes for HPIV and HRSV, *χ*^2^ > 1,000, *P* < 0.001). HRV predominated in spring and autumn; *human parainfluenza virus* (HPIV) exhibited a striking summer peak (17.3%); and HRSV, influenza, and *human metapneumovirus* clustered in winter (HRSV 21.9%). These “Seasonal Characteristics” meaningfully alter pretest probability: summer croup should raise strong suspicion for HPIV, whereas winter bronchiolitis in infants points principally to HRSV (33, 34). Operationally, infection control can be seasonally calibrated—intensifying HPIV and adenovirus precautions in childcare settings during summer, and activating HRSV- and influenza-focused prevention (vaccination campaigns, visitor restrictions, cohorting) ahead of winter, with HRSV prophylaxis timed to anticipate early surges rather than adhering to fixed calendar dates (35, 36). Infection control capacity should therefore be aligned with predictable winter HRSV–influenza peaks, and complemented by educational campaigns that promote hand hygiene, mask use, and ventilation, intensified before the autumn HRV peak and winter viral season (37). Because causation is not proven by association and local variation exists, such seasonal thresholds should be validated locally and embedded in flexible, surveillance-driven protocols.

### 4.3 Age and sex disparities

Pathogen detection showed clear age tropisms. HRSV dominated in infants (27.7%), consistent with waning maternal antibodies and narrow airways predisposing to severe bronchiolitis (38). *MP* was concentrated in school-aged children, reflecting transmission in classroom settings, while HRV and HPIV were most frequent in toddlers and preschoolers. The very low positivity in neonates likely reflects combined maternal antibody protection and reduced community exposure (38). Modest but significant sex differences were observed: HRV, Boca, and HRSV were more common in males, whereas *MP* was marginally more frequent in females. Significant sex disparities were observed for several pathogens, but the absolute differences were minimal (e.g., HRV 24.3% in males vs. 21.0% in females). In a cohort of this size, even trivially small differences can generate significant *P* values. Consequently, the clinical importance of these findings should be interpreted with caution, and they should not be used to substantiate sex-based differences in pathogen susceptibility without further prospective validation. These associations may reflect sex-linked immune variation, behavioral factors, or testing biases and require prospective validation before causal inference (39). In the clinic, age and sex should refine pretest probability—for instance, a wheezing infant in winter is most likely HRSV-infected, whereas a school-age child with persistent cough in autumn warrants consideration of *MP* (40).

### 4.4 Co-infection

Co-infection occurred in 13.6% of cases, peaking in winter, among inpatients, and in toddlers and preschoolers. After accounting for within-influenza subtype co-detections (which may reflect cross-reactivity rather than true co-infection), the most common non-influenza pairs were HRSV–HRV and HRV–HADV. The high rate of co-detection involving HRV and other respiratory pathogens should be interpreted in the context of its known propensity for prolonged and asymptomatic shedding. The presence of HRV nucleic acid alongside another pathogen does not necessarily confirm a causal co-infection that worsens clinical disease status; instead, it may partly reflect background carriage in a subset of patients. Consequently, the true co-infection rate and its clinical significance may be confounded, compromising the accuracy of direct attribution. Future studies incorporating quantitative viral load thresholds or longitudinal symptom correlation are warranted to distinguish asymptomatic carriage from active co-infections. Multiplex PCR provides etiologic breadth but cannot reliably distinguish active co-infection from sequential shedding or residual nucleic acid; results must be interpreted using clinical context, and quantitative Ct values where available, with confirmatory culture or sequencing for unusual combinations (41). Observational evidence suggests that certain dual infections—particularly HRSV with HRV—may carry a higher risk of severe outcomes (42). To maximize clinical utility without driving unnecessary intervention, multiplex molecular diagnostics should be applied selectively and strategically—in hospitalized, severe, or non-responsive cases—thereby reducing unnecessary antibiotic use (especially important given that HRV and HRSV together accounted for 38.1% of all detections) while permitting timely targeted therapy when *MP* is confirmed (43). Such an approach supports antiviral choice, appropriate cohorting, and more accurate prognostication.

## 5 Limitations

This study has several limitations. As a retrospective, single-center analysis, external generalizability is limited. We lacked systematic clinical severity measures, comorbidity data, vaccination status, and treatment/outcome information, preventing correlation of specific pathogens or co-infection with clinical severity. Testing was qualitative; viral load, subtyping, and genotyping were not performed for all pathogens. Finally, potential biases in testing indications and health-seeking behavior over time may affect observed trends. Our 13 respiratory pathogens detected InfA as a separate target from its subtypes (H1N1 and *H3N2*), leading to an overestimation of co-infection counts where these targets overlapped. Therefore, co-infection rates involving influenza viruses should be interpreted with extreme caution. Given the unadjusted *P*-values for multiple comparisons by season/age group, these findings require further validation.

## 6 Implications and future directions

Our findings support targeted pathogen screening based on season and age, which may improve diagnostic efficiency and resource allocation. A key limitation of this study is our inability to distinguish between true HRV infection and asymptomatic colonization, as we relied solely on qualitative PCR testing without clinical symptom correlation or viral load quantification. Given the documented high rate of asymptomatic HRV carriage in pediatric populations, this may have inflated both the positivity rate and the apparent co-infection rate, potentially leading to discrepancies between microbiological findings and actual disease attribution. Our reported co-infection data should therefore be viewed as “co-detection” events, and clinical interpretation requires further individual assessment. Prospective, multicenter studies incorporating clinical outcomes, vaccination data, pathogen genotyping, and serological assessments are needed to clarify the clinical impact of co-infection and the drivers of post-pandemic temporal shifts. Enhanced sentinel surveillance with standardized molecular panels will help optimize prevention (e.g., HRSV prophylaxis, influenza vaccination timing) and clinical management for pediatric populations.

## 7 Conclusions

This large-scale surveillance of 32,125 children from 2023 to 2025 demonstrates a sustained high burden of pediatric respiratory infections, with 63.4% of tested samples positive for at least one pathogen. HRV (22.9%) and HRSV (15.2%) were the dominant pathogens, together accounting for over one-third of all detections, and single infections constituted the majority of cases (49.8%), while co-infections occurred in 13.6%, predominantly as dual infections. The study reveals distinct temporal and population patterns with direct clinical implications: HRSV displayed a pronounced post-pandemic resurgence, peaking at 20.6% in 2025 and concentrated in infants, which underscores the need for flexible, surveillance-driven timing of immunoprophylaxis rather than fixed calendar-based schedules. In contrast, *MP* showed a continuous decline to 1.3%, likely reflecting post-epidemic host depletion, natural cyclicity, and the effect of pre-test antibiotic treatment, but its predilection for school-aged children warrants continued vigilance. Clear seasonal predilections were evident—HPIV peaked in summer, HRSV and influenza in winter—allowing for season-specific refinement of pretest probability and targeted infection control measures. Age tropisms further refine diagnostic suspicion, with HRSV dominating in infants and *MP* and adenovirus in school-aged and preschool children. The observed low positivity rates for influenza and *MP* must be interpreted in light of selection bias introduced by stepwise single-target testing strategies; many positive cases were likely diagnosed before multiplex panel application, meaning these figures do not contradict high community prevalence from unbiased surveillance. Importantly, the high HRV positivity must be viewed with caution due to frequent asymptomatic carriage, and molecular co-detection does not always equate to clinically significant co-infection. Consequently, multiplex respiratory panels should be deployed selectively in severe, hospitalized, or treatment-nonresponsive cases to maximize clinical utility and support antibiotic stewardship. Overall, this contemporary pathogen landscape supports the integration of real-time local surveillance data into clinical algorithms, enabling seasonally adaptive diagnosis, appropriately timed prevention strategies, and judicious antimicrobial use in children.

## Data Availability

The raw data supporting the conclusions of this article will be made available by the authors, without undue reservation.
