# Supplementary material for: Epidemiology, temporal trends and co-infection of 13 respiratory pathogens in 32,125 children: a 3-year retrospective study
Source: Front Pediatr. 2026 Aug 14;14:1884107. doi: 10.3389/fped.2026.1884107 (PMC13522108; doi:10.3389/fped.2026.1884107)
Supplement: Supplementary file 1 [file Supplementaryfile1.docx]

Figure S1. Official ethical approval certificate issued by the Ethics Committee

Table S1. Annual positive and negative detection rates of 13 respiratory pathogens among children

| Variables | Total  (n = 32125) | 2023  (n = 8073) | 2024  (n = 13701) | 2025  (n = 10351) | p | statistic |
| --- | --- | --- | --- | --- | --- | --- |
| **HRV, n (%)** |  |  |  |  | < 0.001 | 249.39 |
| Negative | 24784 (77.1) | 6742 (83.5) | 10236 (74.7) | 7806 (75.4) |  |  |
| Positive | 7341 (22.9) | 1331 (16.5) | 3465 (25.3) | 2545 (24.6) |  |  |
| **Boca, n (%)** |  |  |  |  | < 0.001 | 76.87 |
| Negative | 31563 (98.3) | 7859 (97.3) | 13554 (98.9) | 10150 (98.1) |  |  |
| Positive | 562 (1.7) | 214 (2.7) | 147 (1.1) | 201 (1.9) |  |  |
| **MP, n (%)** |  |  |  |  | < 0.001 | 857.47 |
| Negative | 29858 (92.9) | 7104 (88.0) | 12542 (91.5) | 10212 (98.7) |  |  |
| Positive | 2267 (7.1) | 969 (12.0) | 1159 (8.5) | 139 (1.3) |  |  |
| **HPIV, n (%)** |  |  |  |  | < 0.001 | 43.15 |
| Negative | 29400 (91.5) | 7437 (92.1) | 12379 (90.4) | 9584 (92.6) |  |  |
| Positive | 2725 (8.5) | 636 (7.9) | 1322 (9.6) | 767 (7.4) |  |  |
| **HCoV, n (%)** |  |  |  |  | < 0.001 | 15.58 |
| Negative | 31478 (98.0) | 7877 (97.6) | 13471 (98.3) | 10130 (97.9) |  |  |
| Positive | 647 (2.0) | 196 (2.4) | 230 (1.7) | 221 (2.1) |  |  |
| **HRSV, n (%)** |  |  |  |  | < 0.001 | 400.16 |
| Negative | 27239 (84.8) | 6850 (84.9) | 12166 (88.8) | 8223 (79.4) |  |  |
| Positive | 4886 (15.2) | 1223 (15.1) | 1535 (11.2) | 2128 (20.6) |  |  |
| **H3N2, n (%)** |  |  |  |  | < 0.001 | 175.82 |
| Negative | 31566 (98.3) | 7817 (96.8) | 13597 (99.2) | 10152 (98.1) |  |  |
| Positive | 559 (1.7) | 256 (3.2) | 104 (0.8) | 199 (1.9) |  |  |
| **InfA, n (%)** |  |  |  |  | 0.018 | 8.09 |
| Negative | 30830 (96.0) | 7706 (95.5) | 13185 (96.2) | 9939 (96.0) |  |  |
| Positive | 1295 (4.0) | 367 (4.5) | 516 (3.8) | 412 (4.0) |  |  |
| **InfA.H1N1.2009, n (%)** |  |  |  |  | < 0.001 | 62.59 |
| Negative | 31407 (97.8) | 7966 (98.7) | 13300 (97.1) | 10141 (98.0) |  |  |
| Positive | 718 (2.2) | 107 (1.3) | 401 (2.9) | 210 (2.0) |  |  |
| **HMPV, n (%)** |  |  |  |  | < 0.001 | 78.56 |
| Negative | 30776 (95.8) | 7610 (94.3) | 13136 (95.9) | 10030 (96.9) |  |  |
| Positive | 1349 (4.2) | 463 (5.7) | 565 (4.1) | 321 (3.1) |  |  |
| **HADV, n (%)** |  |  |  |  | < 0.001 | 1125.18 |
| Negative | 29770 (92.7) | 7908 (98.0) | 11931 (87.1) | 9931 (95.9) |  |  |
| Positive | 2355 (7.3) | 165 (2.0) | 1770 (12.9) | 420 (4.1) |  |  |
| **Ch, n (%)** |  |  |  |  | < 0.001 | 17.04 |
| Negative | 31922 (99.4) | 8045 (99.7) | 13612 (99.4) | 10265 (99.2) |  |  |
| Positive | 203 (0.6) | 28 (0.3) | 89 (0.6) | 86 (0.8) |  |  |
| **InfB, n (%)** |  |  |  |  | < 0.001 | 152.49 |
| Negative | 31779 (98.9) | 7940 (98.4) | 13493 (98.5) | 10346 (100) |  |  |
| Positive | 346 (1.1) | 133 (1.6) | 208 (1.5) | 5 (0.0) |  |  |

Note：Percentages were rounded to one decimal place; column sums may not equal 100% due to rounding. Human rhinovirus (HRV), human bocavirus (Boca), Mycoplasma pneumoniae (MP), human parainfluenza virus (HPIV), human coronavirus (HCoV), human respiratory syncytial virus (HRSV), influenza A virus subtype H3N2 (H3N2), influenza A virus (InfA), 2009 pandemic influenza A (H1N1) virus (InfA.H1N1.2009), human metapneumovirus (HMPV), human adenovirus (HADV), Chlamydia species (Ch), and influenza B virus (InfB). newborns (0–28 days), infants (29 days to <1 year), toddlers (1 to <3 years), preschoolers (3 to <6 years), school-aged children (6 to <12 years), and adolescents (12–18 years).

Table S2. Distribution patterns of various co-infection modes

| **Variables** | **Total**  **(n = 32125)** | **No**  **(n = 11744)** | **Single**  **(n = 16003)** | **Double**  **(n = 3919)** | **Triple**  **(n = 426)** | **Quadruple**  **(n = 31)** | **Quintuple**  **(n = 2)** | **P** | **Statistic** |
| --- | --- | --- | --- | --- | --- | --- | --- | --- | --- |
| Gender, n (%) |  |  |  |  |  |  |  | <0.001 | Fisher’s exact test |
| Female | 13851 (43.1) | 5318 (45.3) | 6736 (42.1) | 1620 (41.3) | 167 (39.2) | 10 (32.3) | 0 (0.0) |  |  |
| Male | 18274 (56.9) | 6426 (54.7) | 9267 (57.9) | 2299 (58.7) | 259 (60.8) | 21 (67.7) | 2 (100.0) |  |  |
| Year, n (%) |  |  |  |  |  |  |  | <0.001 | Fisher’s exact test |
| 2023 | 8073 (25.1) | 3256 (27.7) | 3672 (22.9) | 1028 (26.2) | 108 (25.4) | 9 (29.0) | 0 (0.0) |  |  |
| 2024 | 13701 (42.6) | 4507 (38.4) | 7116 (44.5) | 1855 (47.3) | 208 (48.8) | 14 (45.2) | 1 (50.0) |  |  |
| 2025 | 10351 (32.2) | 3981 (33.9) | 5215 (32.6) | 1036 (26.4) | 110 (25.8) | 8 (25.8) | 1 (50.0) |  |  |
| Season, n (%) |  |  |  |  |  |  |  | <0.001 | Fisher’s exact test |
| Spring | 7713 (24.0) | 2817 (24.0) | 3948 (24.7) | 880 (22.5) | 67 (15.7) | 1 (3.2) | 0 (0.0) |  |  |
| Summer | 7559 (23.5) | 3080 (26.2) | 3603 (22.5) | 804 (20.5) | 70 (16.4) | 1 (3.2) | 1 (50.0) |  |  |
| Fall | 8204 (25.5) | 2880 (24.5) | 4217 (26.4) | 1003 (25.6) | 96 (22.5) | 8 (25.8) | 0 (0.0) |  |  |
| Winter | 8649 (26.9) | 2967 (25.3) | 4235 (26.5) | 1232 (31.4) | 193 (45.3) | 21 (67.7) | 1 (50.0) |  |  |
| Agegroups, n (%) |  |  |  |  |  |  |  | <0.001 | Fisher’s exact test |
| Newborns | 1520 ( 4.7) | 1134 (9.7) | 361 (2.3) | 25 (0.6) | 0 (0.0) | 0 (0.0) | 0 (0.0) |  |  |
| Infants | 7505 (23.4) | 2648 (22.5) | 3916 (24.5) | 840 (21.4) | 97 (22.8) | 4 (12.9) | 0 (0.0) |  |  |
| Toddlers | 7781 (24.2) | 1980 (16.9) | 4510 (28.2) | 1146 (29.2) | 133 (31.2) | 11 (35.5) | 1 (50.0) |  |  |
| Preschoolers | 7424 (23.1) | 2117 (18.0) | 4093 (25.6) | 1091 (27.8) | 112 (26.3) | 11 (35.5) | 0 (0.0) |  |  |
| School-aged children | 6360 (19.8) | 2834 (24.1) | 2736 (17.1) | 713 (18.2) | 75 (17.6) | 2 (6.5) | 0 (0.0) |  |  |
| Adolescents | 1535 ( 4.8) | 1031 (8.8) | 387 (2.4) | 104 (2.7) | 9 (2.1) | 3 (9.7) | 1 (50.0) |  |  |
| Visit type, n (%) |  |  |  |  |  |  |  | <0.001 | Fisher’s exact test |
| EP | 9591 (29.9) | 2110 (18.0) | 5702 (35.6) | 1583 (40.4) | 182 (42.7) | 13 (41.9) | 1 (50.0) |  |  |
| IP | 19984 (62.2) | 8960 (76.3) | 8919 (55.7) | 1900 (48.5) | 188 (44.1) | 16 (51.6) | 1 (50.0) |  |  |
| OP | 2550 ( 7.9) | 674 (5.7) | 1382 (8.6) | 436 (11.1) | 56 (13.1) | 2 (6.5) | 0 (0.0) |  |  |

Note：Percentages were rounded to one decimal place; column sums may not equal 100% due to rounding.
